# Supplementary material for: Distinct Subtypes of Hepatorenal Syndrome and Associated Outcomes as Identified by Machine Learning Consensus Clustering
Source: Diseases. 2023 Jan 27;11(1):18. doi: 10.3390/diseases11010018 (PMC9944494; doi:10.3390/diseases11010018)
Supplement: Supplementary file 1 [file diseases-11-00018-s001.zip › diseases-2109680-supplementary.pdf]

eMethods

Cluster derivation

We applied an unsupervised ML approach to develop clinical phenotypes of HRS patients in the NIS/HCUP database by conducting unsupervised consensus clustering.[1] We performed consensus clustering analysis on the whole study population. We initially assessed the distribution and missingness in phenotyping variables. Subsequently, non-normal data were z-score normalized. We subsequently applied clustering using the consensus cluster algorithm. The algorithm begins by subsampling a proportion of items and a proportion of features from a data matrix. Each subsample is then partitioned into up to groups (k) by a user-specified clustering algorithm. This process is repeated for a specified number of times. Pairwise consensus values, defined as 'the proportion of clustering runs in which two items are grouped together, are calculated and stored in a consensus matrix (CM) for each cluster. Clustering settings used were as follows: maximum number of clusters, 10; number of iterations, 100; subsampling fraction, 0.8; clustering algorithm, , K-means; Euclidean distance).[1] The number of potential clusters ranges from 2 to 10, to avoid producing an excessive number of clusters that would not be clinical useful. Pairwise consensus values, defined as 'the proportion of clustering runs in which two items are [grouped] together[1], are calculated and stored in a CM for each k. Then for each k, a final agglomerative hierarchical consensus clustering using distance of 1−consensus values is completed and pruned to k groups, which are called consensus clusters.

The clustering algorithm is to maximize the potential number of clusters while maintaining high cluster consensus. The optimal number of clusters was determined by examining the CM heat map, cumulative distribution function, and cluster-consensus plots with the within-cluster consensus scores.[2,3] The within-cluster consensus score, ranging between 0 and 1, is defined as the average consensus value for all pairs of individuals belonging to the

same cluster.[3] A value closer to one indicates better cluster stability.[3] To examine the cluster profile, we calculated and graphically displayed the standardized mean differences of the variables between each cluster and the overall study population. Calculation of the standardized difference of each parameter used the cutoff of ±0.3 to show subgroup features with the key features for each cluster [4-15].

All cluster derivation analyses were performed using R, version 4.0.3 (RStudio, Inc., Boston, MA; http://www.rstudio.com/), with the packages of ConsensusClusterPlus (version 1.46.0)[3]. All analyses were two-tailed, and P value < .05 was considered statistically significant.

Figure S1. Consensus matrix heat map (k = 2) depicting consensus values on a white to blue color scale of each cluster

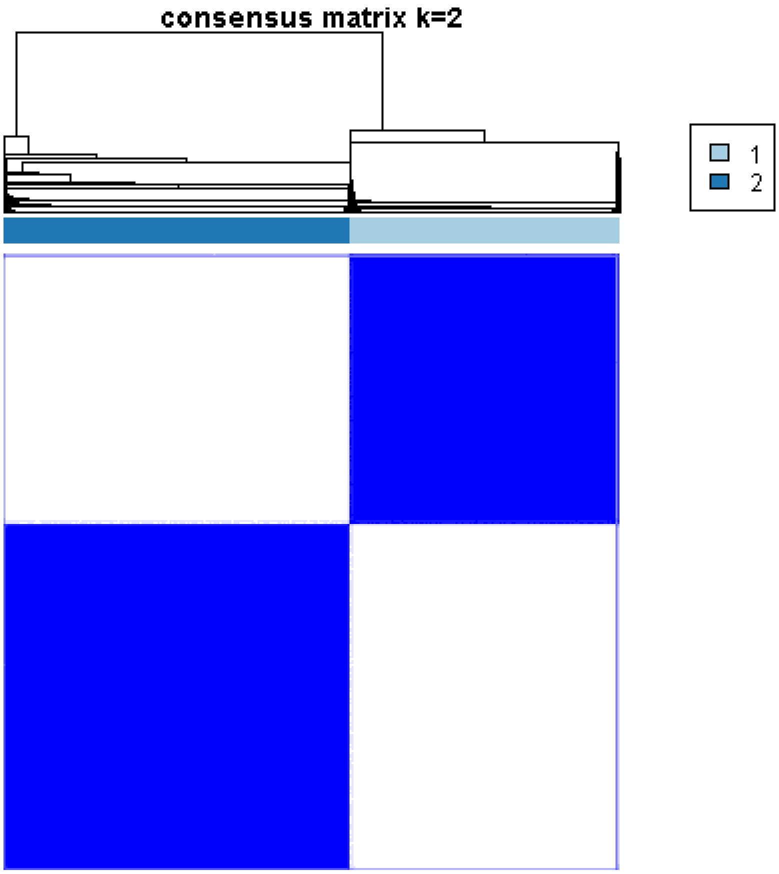

Figure S2. Consensus matrix heat map (k = 3) depicting consensus values on a white to blue color scale of each cluster

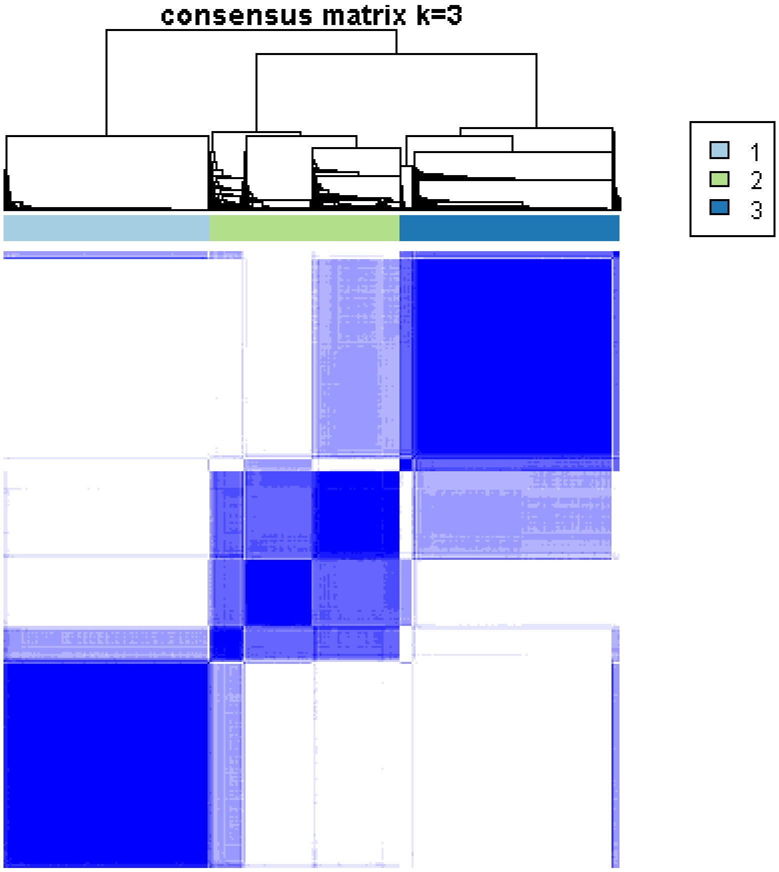

Figure S3. Consensus matrix heat map (k = 4) depicting consensus values on a white to blue color scale of each cluster

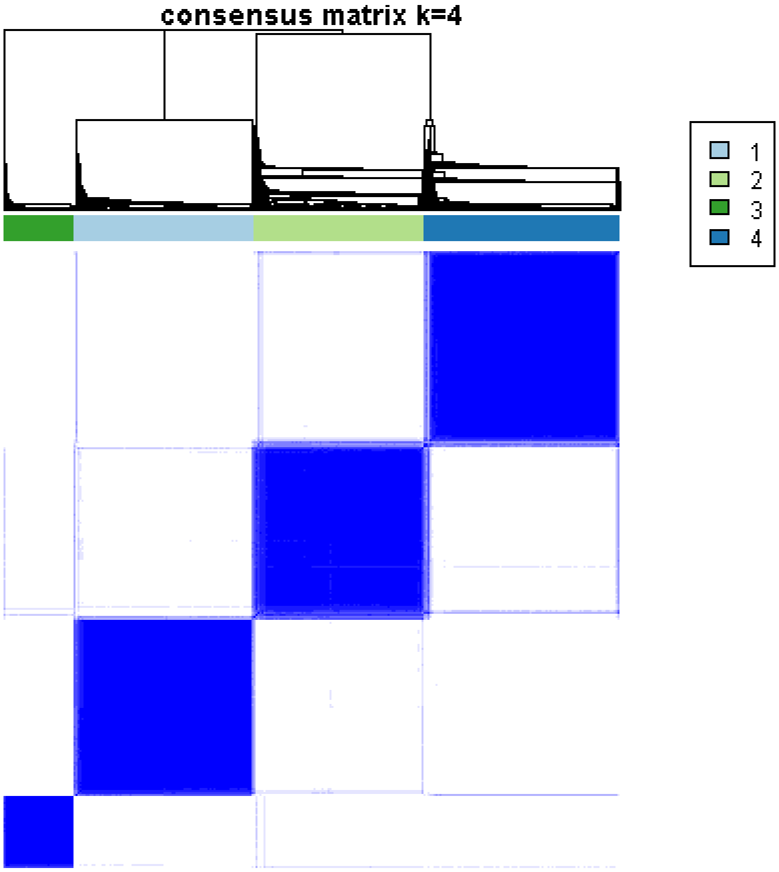

Figure S4. Consensus matrix heat map (k = 5) depicting consensus values on a white to blue color scale of each cluster

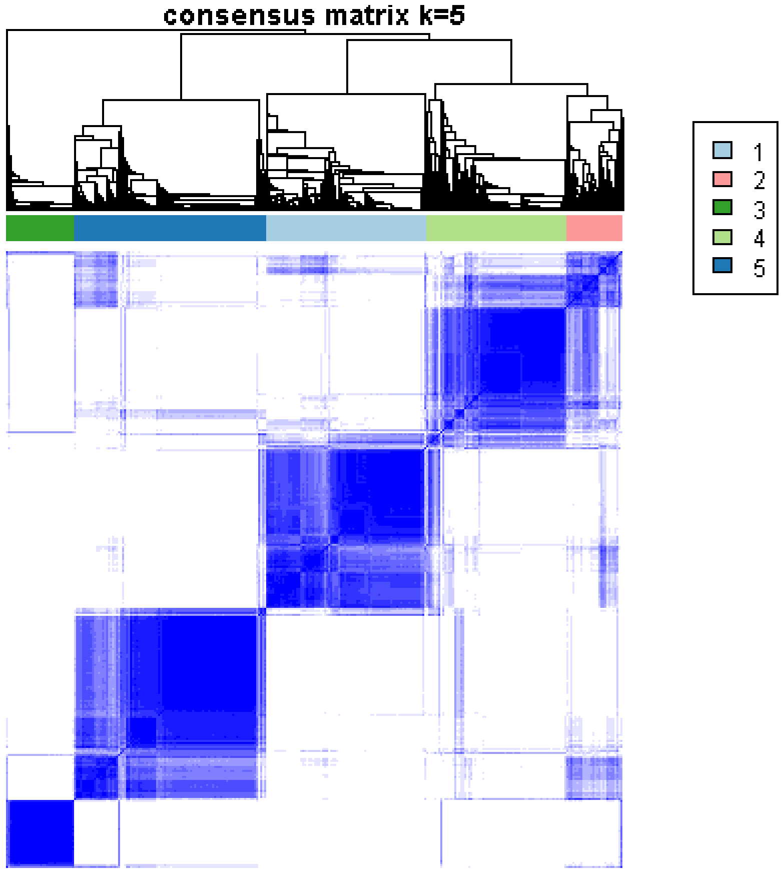

Figure S5. Consensus matrix heat map (k = 6) depicting consensus values on a white to blue color scale of each cluster

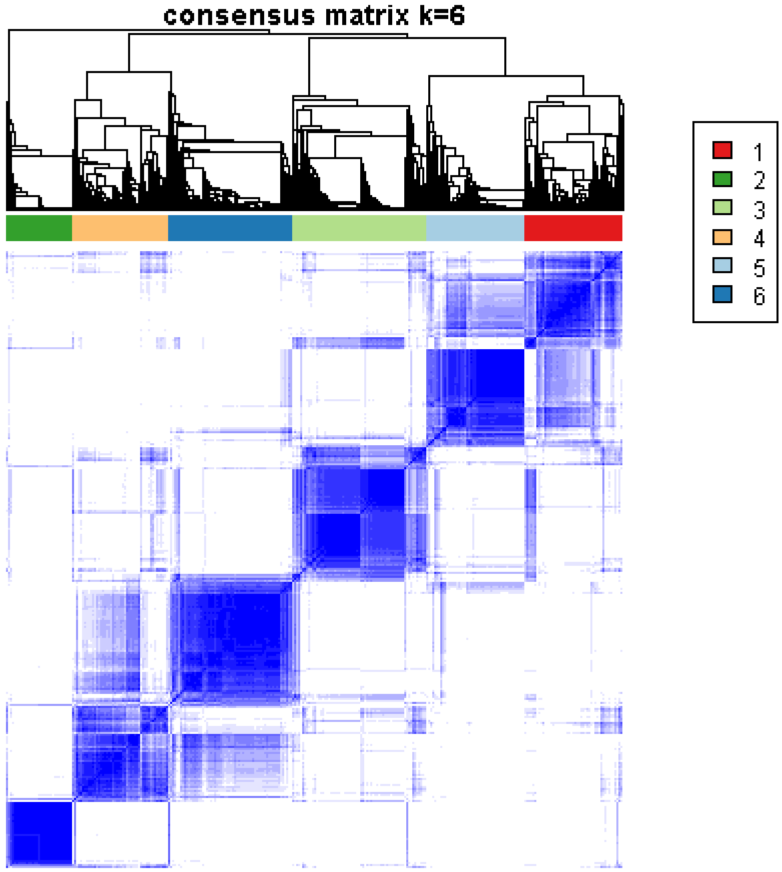

Figure S6. Consensus matrix heat map (k = 7) depicting consensus values on a white to blue color scale of each cluster

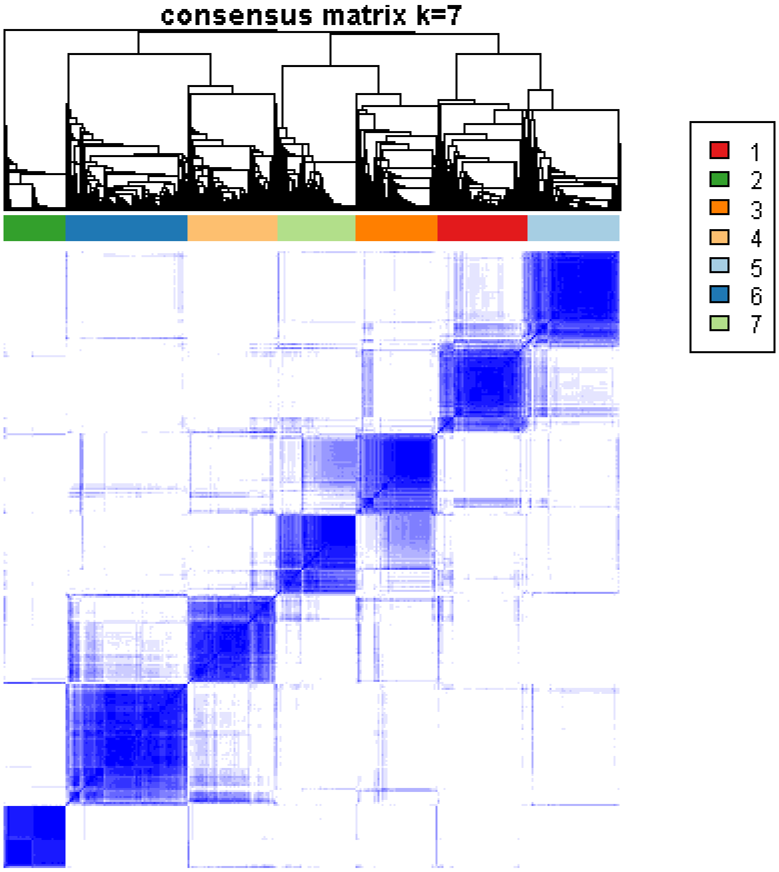

Figure S7. Consensus matrix heat map (k = 8) depicting consensus values on a white to blue color scale of each cluster

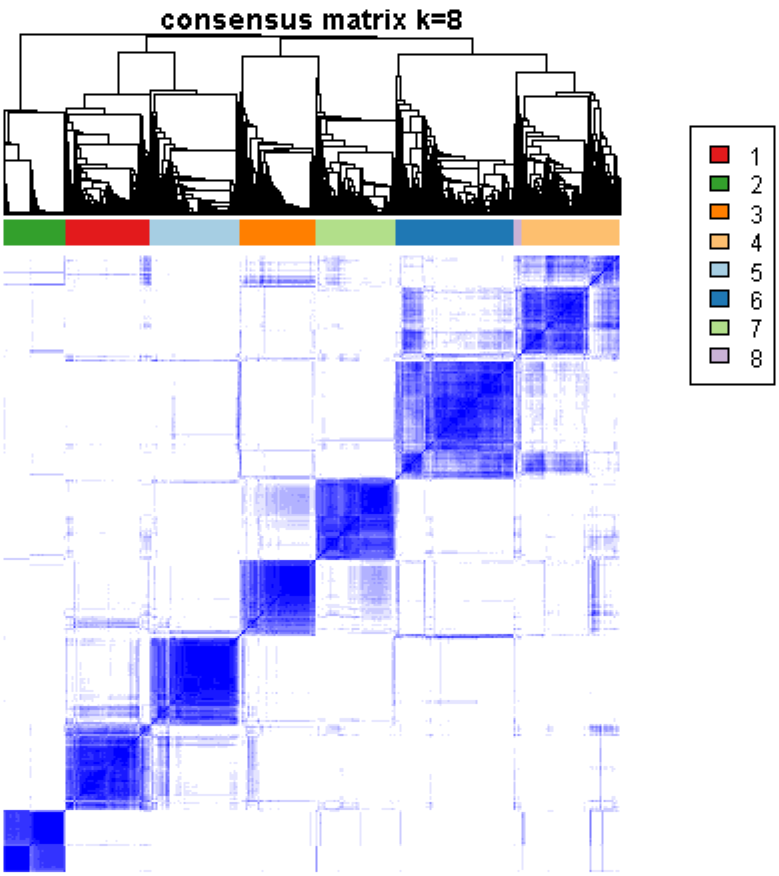

Figure S8. Consensus matrix heat map (k = 9) depicting consensus values on a white to blue color scale of each cluster

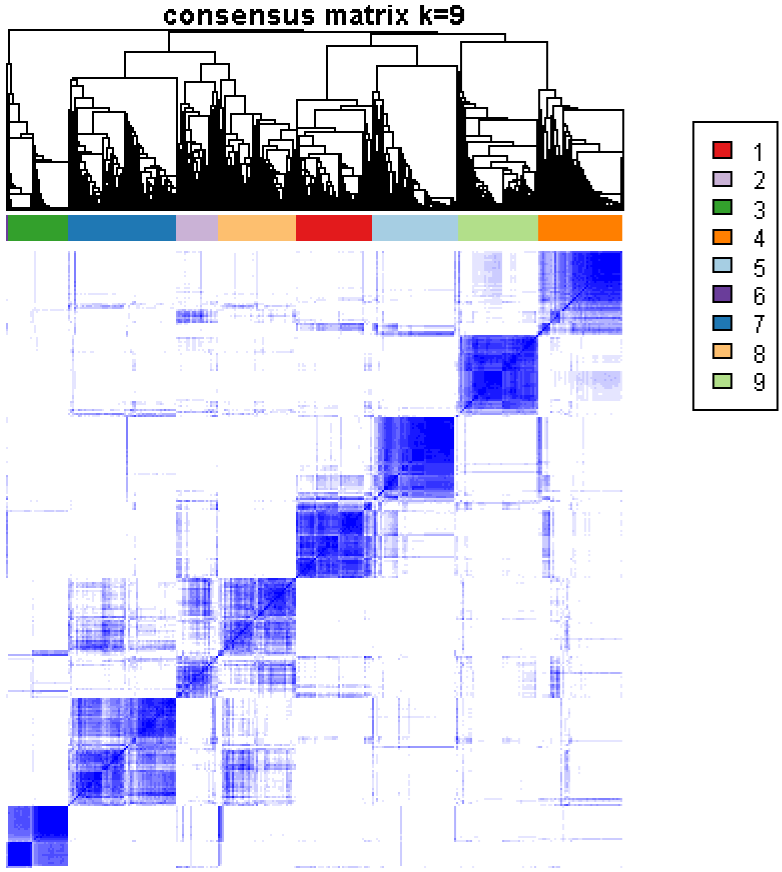

Figure S9. Consensus matrix heat map (k = 10) depicting consensus values on a white to blue color scale of each cluster

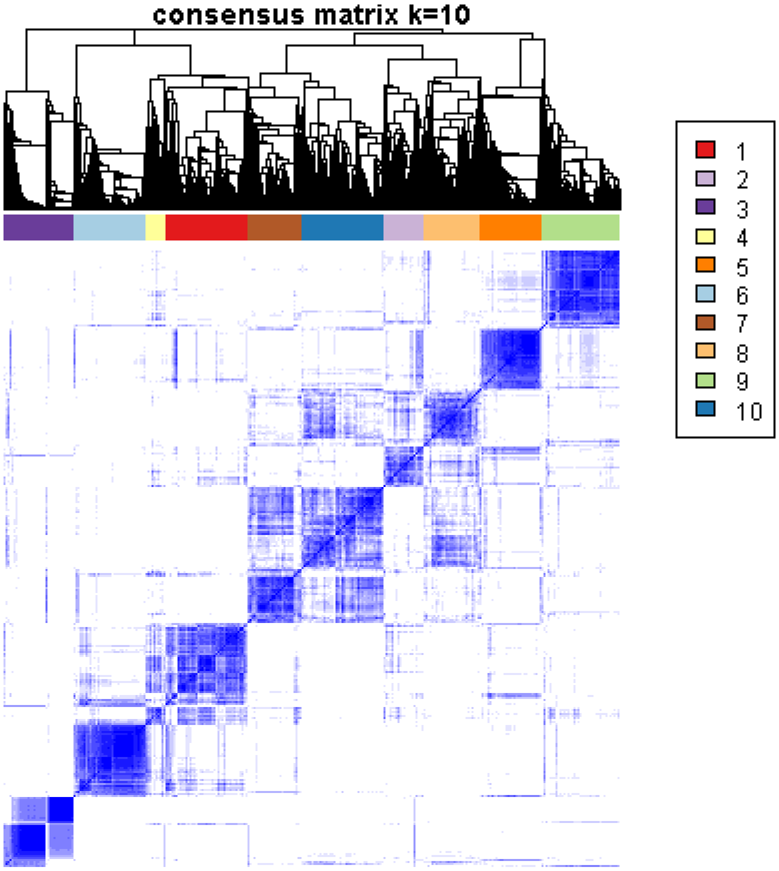

References

1. Monti, S.; Tamayo, P.; Mesirov, J.; Golub, T. Consensus clustering: a resampling-based method for class discovery and visualization of gene expression microarray data. *Machine learning* **2003**, *52*, 91-118.
2. Şenbabaoğlu, Y.; Michailidis, G.; Li, J.Z. Critical limitations of consensus clustering in class discovery. *Sci Rep* **2014**, *4*, 6207, doi:10.1038/srep06207.
3. Wilkerson, M.D.; Hayes, D.N. ConsensusClusterPlus: a class discovery tool with confidence assessments and item tracking. *Bioinformatics* **2010**, *26*, 1572-1573.
4. Pattharanitima, P.; Thongprayoon, C.; Petnak, T.; Srivali, N.; Gembillo, G.; Kaewput, W.; Chesdachai, S.; Vallabhajosyula, S.; O'Corragain, O.A.; Mao, M.A., et al. Machine Learning Consensus Clustering Approach for Patients with Lactic Acidosis in Intensive Care Units. *J Pers Med* **2021**, *11*, doi:10.3390/jpm11111132.
5. Thongprayoon, C.; Dumancas, C.Y.; Nissaisorakarn, V.; Keddis, M.T.; Kattah, A.G.; Pattharanitima, P.; Petnak, T.; Vallabhajosyula, S.; Garovic, V.D.; Mao, M.A., et al. Machine Learning Consensus Clustering Approach for Hospitalized Patients with Phosphate Derangements. *J Clin Med* **2021**, *10*, doi:10.3390/jcm10194441.
6. Thongprayoon, C.; Hansrivijit, P.; Mao, M.A.; Vaitla, P.K.; Kattah, A.G.; Pattharanitima, P.; Vallabhajosyula, S.; Nissaisorakarn, V.; Petnak, T.; Keddis, M.T., et al. Machine Learning Consensus Clustering of Hospitalized Patients with Admission Hyponatremia. *Diseases* **2021**, *9*, doi:10.3390/diseases9030054.
7. Thongprayoon, C.; Jadlowiec, C.C.; Kaewput, W.; Vaitla, P.; Mao, S.A.; Mao, M.A.; Leeaphorn, N.; Qureshi, F.; Pattharanitima, P.; Qureshi, F., et al. Distinct Phenotypes of Kidney Transplant Recipients in the United States with Limited Functional Status as Identified through Machine Learning Consensus Clustering. *J Pers Med* **2022**, *12*, doi:10.3390/jpm12060859.
8. Thongprayoon, C.; Kattah, A.G.; Mao, M.A.; Keddis, M.T.; Pattharanitima, P.; Vallabhajosyula, S.; Nissaisorakarn, V.; Erickson, S.B.; Dillon, J.J.; Garovic, V.D., et al. Distinct phenotypes of hospitalized patients with hyperkalemia by machine learning consensus clustering and associated mortality risks. *Qjm* **2022**, *115*, 442-449, doi:10.1093/qjmed/hcab194.
9. Thongprayoon, C.; Mao, M.A.; Kattah, A.G.; Keddis, M.T.; Pattharanitima, P.; Erickson, S.B.; Dillon, J.J.; Garovic, V.D.; Cheungpasitporn, W. Subtyping hospitalized patients with hypokalemia by machine learning consensus clustering and associated mortality risks. *Clin Kidney J* **2022**, *15*, 253-261, doi:10.1093/ckj/sfab190.
10. Thongprayoon, C.; Mao, M.A.; Keddis, M.T.; Kattah, A.G.; Chong, G.Y.; Pattharanitima, P.; Nissaisorakarn, V.; Garg, A.K.; Erickson, S.B.; Dillon, J.J., et al. Hypernatremia subgroups among hospitalized patients by machine learning consensus clustering with different patient survival. *J Nephrol* **2022**, *35*, 921-929, doi:10.1007/s40620-021-01163-2.
11. Thongprayoon, C.; Mao, S.A.; Jadlowiec, C.C.; Mao, M.A.; Leeaphorn, N.; Kaewput, W.; Vaitla, P.; Pattharanitima, P.; Tangpanithandee, S.; Krisanapan, P., et al. Machine Learning Consensus Clustering of Morbidly Obese Kidney Transplant Recipients in the United States. *J Clin Med* **2022**, *11*, doi:10.3390/jcm11123288.
12. Thongprayoon, C.; Nissaisorakarn, V.; Pattharanitima, P.; Mao, M.A.; Kattah, A.G.; Keddis, M.T.; Dumancas, C.Y.; Vallabhajosyula, S.; Petnak, T.; Erickson, S.B., et al. Subtyping Hyperchloremia among Hospitalized Patients by Machine Learning Consensus Clustering. *Medicina (Kaunas)* **2021**, *57*, doi:10.3390/medicina57090903.
13. Thongprayoon, C.; Sy-Go, J.P.T.; Nissaisorakarn, V.; Dumancas, C.Y.; Keddis, M.T.; Kattah, A.G.; Pattharanitima, P.; Vallabhajosyula, S.; Mao, M.A.; Qureshi, F., et al. Machine Learning

Consensus Clustering Approach for Hospitalized Patients with Dysmagnesemia. *Diagnostics (Basel)* **2021**, *11*, doi:10.3390/diagnostics11112119.
14. Thongprayoon, C.; Vaitla, P.; Jadlowiec, C.C.; Leeaphorn, N.; Mao, S.A.; Mao, M.A.; Pattharanitima, P.; Bruminhent, J.; Khoury, N.J.; Garovic, V.D., et al. Use of Machine Learning Consensus Clustering to Identify Distinct Subtypes of Black Kidney Transplant Recipients and Associated Outcomes. *JAMA Surg* **2022**, *157*, e221286, doi:10.1001/jamasurg.2022.1286.
15. Thongprayoon, C.; Vaitla, P.; Nissaisorakarn, V.; Mao, M.A.; Genovez, J.L.Z.; Kattah, A.G.; Pattharanitima, P.; Vallabhajosyula, S.; Keddis, M.T.; Qureshi, F., et al. Clinically Distinct Subtypes of Acute Kidney Injury on Hospital Admission Identified by Machine Learning Consensus Clustering. *Med Sci (Basel)* **2021**, *9*, doi:10.3390/medsci9040060.
